# Diagnostic Challenge and Neuromuscular Junction Contribution to ALS Pathogenesis

**DOI:** 10.3389/fneur.2019.00068

**Published:** 2019-02-06

**Authors:** Maria-Letizia Campanari, Annis-Rayan Bourefis, Edor Kabashi

**Affiliations:** ^1^Sorbonne Université, Université Pierre et Marie Curie, Université de Paris 06, Unité Mixte 75, Institut National de la Santé et de la Recherche Médicale (INSERM) Unité 1127, Centre National de la Recherche Scientifique, Unité Mixte de Recherche 7225 Institut du Cerveau et de la Moelle Épinière, Paris, France; ^2^Imagine Institute, INSERM Unité 1163, Paris Descartes Université, Paris, France

**Keywords:** amyotrophic lateral sclerosis (ALS), axonopathy, neuromuscular junction (NMJ), dying back hypothesis, ALS-mimic diseases

## Abstract

Amyotrophic lateral sclerosis (ALS) represents the major adult-onset motor neuron disease. Both human and animal studies reveal the critical implication of muscle and neuromuscular junctions (NMJs) in the initial phase of this disease. Despite the common efforts, ALS diagnosis remains particularly challenging since many other disorders can overlap yielding similar clinical phenotypic features. A combination of further research on the NMJ parameters that are specific for this disease and laboratory tests are crucial for the early determination of specific changes in the muscle, as well as in motor neuron and the prediction of ALS progression. Also, it could provide a powerful tool in the discrimination of particular ALS and ALS-mimic cases and increase the efficacy of therapeutic treatments.

## Amyotrophic Lateral Sclerosis

Amyotrophic Lateral Sclerosis (ALS) is a disease characterized by a progressive degeneration of upper motor neurons (MNs) in the motor cortex and lower motor neurons in the brainstem and the spinal cord. The death of these neurons leads to spasticity, weakness, and atrophy of the muscles, progressing to paralysis. The incidence of ALS in Europe is 2–16 per 100,000 each year (1), with respiratory failure being the predominant mode of death in patients within 3 years of diagnosis (2). The onset of the disease occurs prevalently during adulthood (peak age of 58–63 years) (3), though with a small proportion of early-onset disease in certain patients (before 35 years of age). ALS also shares neuropathological and genetic features with another neurodegenerative disorder, frontotemporal dementia (FTD) (4, 5), with many ALS patients showing some cognitive or behavioral changes. This has led to consider ALS and FTD as the ends of the same spectrum of disease (6).

Although the majority of ALS cases occur sporadically (sALS), there is a Mendelian inheritance in about 10% of the cases (familial ALS, fALS), mainly in an autosomal, dominant fashion (7). The two are clinically indistinguishable and a variety of genetic defects in more than 20 genetic loci have been linked with the ALS phenotype (8), with new genes constantly being identified in subsets of ALS patients (9–11). Four major genes which mutations are known to cause ALS are the f ollowing: chromosome 9 open reading frame 72 (C9orf72), superoxide dismutase 1 (SOD1), transactive response DNA-binding protein (TARDBP) and fused in sarcoma (FUS) (12–15). C9orf72 has an important role in membrane trafficking and autophagy (16), and SOD1 primary function is thought to be as a cytosolic and mitochondrial antioxidant enzyme, converting superoxide to molecular oxygen and hydrogen peroxide (17). TARDBP and FUS encode nucleic acid-binding proteins that reside in the nucleus, and are involved in multiple aspects of RNA processing, such as transcription and splicing [reviewed in (18)].

## NMJ Involvement in ALS

Despite the progress in our understanding of the molecular pathogenesis linked to these genes, it is still unclear where the motor neuron dysfunction begins and the extrinsic factors that accelerate motor neuron degeneration. This led to the consideration of ALS as either a *dying forward* process that proposes an anterograde degeneration of motor neurons by glutamate excitotoxicity from the cortex, or a *dying back* phenomenon in which motor neuron degeneration starts distally at the nerve terminal or at the neuromuscular junction (NMJ) and progresses toward the cell body (3, 19). The NMJ is a tripartite synapse composed by the presynaptic motor neuron, the postsynaptic muscle and the synapse-associated glial cells (terminal Schwann cells, TSC) and allows the transmission of action potentials from motor neurons to muscles [reviewed in (20)]. In this complex structure, besides motor neuron degeneration, glial cells, and muscle fibers play also a major role in ALS onset and progression.

The muscle contribution in ALS development, through NMJs disassembly, is still a matter of debate. Nonetheless, increasing evidence points to the critical role of NMJ defects in the early stage of the disease in ALS patients [reviewed in (21)] and a variety of animal models have permitted important advances into exploring this hypothesis.

The human SOD1^G93A^ transgenic mouse, the first and most studied ALS model, is the one that has yielded the majority of information about the muscular deficits in ALS (22). Spatiotemporal analysis of NMJs in SOD1^G93A^ mouse revealed end-plates denervation before the appearance of clinical symptoms and neuron cell body loss (23), with the fast-fatigable synapses being more vulnerable to denervation (24). Because of its high expression in ALS muscle biopsies, the neurite outgrowth inhibitor Nogo-A was proposed as a factor responsible for motor nerve terminals repulsion and destabilization at the NMJ at very early asymptomatic stages (25, 26). This hypothesis was then confirmed in SOD1^G93A^ mouse model, where genetic ablation of Nogo-A in muscle led to marked reduction of muscle denervation and prolonged survival (27). Morphological observation of NMJs in SOD1^G93A^ also contributed to reinforce the dying back hypothesis, showing more detailed NMJ alterations prior to functional symptom onset (28). A detailed overview of the findings concerning neuromuscular defects in the SOD1^G93A^ mouse model has been reviewed by Dupuis and colleagues (22).

Despite the predominant use of rodent models for studying pathomechanisms and potential therapeutic targets in ALS, the use of smaller animal models, like *Drosophila melanogaster* and zebrafish (*Danio rerio*), is continually increasing. Their advantages lie in their fast development allowing quick generation of lines, their availability and the ease in manipulating gene expression and in drug screening. In drosophila, studies showed locomotor defects, reduced life span, and anatomical defects at the NMJ, causing impairments in synaptic transmission, in loss and gain of function models of TARDBP (29, 30). Similar results were found for FUS. Gene deficiency and overexpression of FUS in Drosophila models caused decreased synaptic transmission, reduced number of presynaptic active zones, altered postsynaptic glutamate receptor subunit composition at the NMJ, motor neuron degeneration and impaired motor behavior (31, 32). Zebrafish studies have highlighted gain and loss of function mechanisms for TARDBP and FUS, demonstrating shorter axonal projections from motor neurons, premature and excessive branching, impaired synaptic transmission at the NMJ leading to swimming defects (33–35). C9orf72 gene has also been modeled in zebrafish in a loss-of-function model that displayed behavioral and cellular deficits related to locomotion (36). For more details about the different models, all the ALS gene mutations that have been modeled are summarized in a recent review by Van Damme et al. (37).

Altogether, fundamental research supports the crucial role that NMJ could play in ALS pathogenesis and its possible employment as efficient early marker of the disease.

## ALS Diagnostic Challenges

The difficulty to diagnose ALS resides mainly in the existence of several mimic syndromes, unrelated to ALS but which present similar clinical features (38, 39).

Motor neuron diseases (MNDs) are classified in four main groups in which ALS represents the most common form (Table 1). Although these diseases affect people in different ways, they share several symptoms due to motor neuron loss of function. All of them present progressive weakening of skeletal muscles, which eventually affects the ability to speak, swallow and breathe. ALS diagnosis is even more difficult if we add to the list other neurological conditions unrelated to MNDs which can mimic its early symptoms. Moreover, increasing evidences point to a possible direct implication of muscle in the early stage of the disease, adding myopathies to the list of ALS-mimic pathologies (Table 1).

**Table 1:** General overview of neuromuscular diseases and ALS-mimic pathologies.

**MOTOR NEURON DISEASES (MND)**	
Amyotrophic Lateral Sclerosis (ALS)	
Primary Lateral Scerosis (PLS)	
Progressive Muscular Atrophy (PMA)	
Progressive Bulbar Palsy (PBP)	
**OTHER NEUROLOGICAL CONDITIONS THAT CAN MIMIC ALS**	
Mithochondrial Disorder (MID)	
Psedobulbar Palsy	
Spinal Muscular atrophy (SMA)	
Primary lateral sclerosis (some subtupes not related to ALS)	
Progressive spinal muscular atrophy (some subtype not related to ALS)	
Spinobulbar muscular atrophy (SBMA or Kennedy's disease)	
Autoimmune Syndromes Monoclonal	
Myopathies	
Cachectic myopathy	
Polymyositis Sarcoid myositis	
Carcinoid myopathy	
Nemaline myopathy	
Inflammatory myopathies	Polymyositis (PM)
	Dermatomyositis	
	Inclusion-body myositis (IBM)	

*Neuromuscular Disorders (NMD) implicate deficits and degeneration of nerves (motor and sensory neurons) and muscles (skeletal muscles) of the central and peripheral nervous system, leading to muscles weaken and waste away (atrophy). NMDs are classified in 4 categories, with Amyotrophic lateral sclerosis representing the main one. ALS-mimic pathologies is a vast group of diseases characterized by weakness and wasting away of muscle tissue, with or without the breakdown of nerve tissue, thus mimicking ALS symptoms. Currently, no cure exists for NMDs and the treatments aim to relieve the symptoms and delay disease progression*.

Standard diagnostic criteria for ALS have been established in 1991 [El Escorial criteria [EEC] (40)] and were revised in 1997 [AirlieHouse criteria [AHC] or El Escorial Revisited (41)]. Even though the essential requirements for ALS diagnosis were defined by these criteria, many neurologists and neuromuscular clinicians were missing the diagnosis, proving the low clinical accuracy of these diagnostic roles (42).

In 2008, electrodiagnostic studies, known as the Awaji criteria (43), were included in the clinical procedure to allow earlier and more accurate assessment of ALS diagnosis. However, the application of those sets of defined features are still insufficient to rule out other similar and related diseases (44, 45).

### Methods for Diagnosis

Although the main ALS evaluation remains the clinical one, laboratory testing, based on advanced techniques of electrodiagnosis, neuroimaging, immunobiochemistry, and neurogenetics, is required for accurate ALS diagnosis.

Tests to rule out other neuromuscular conditions may include:

#### Electromyogram (EMG)

**Figure 1:**
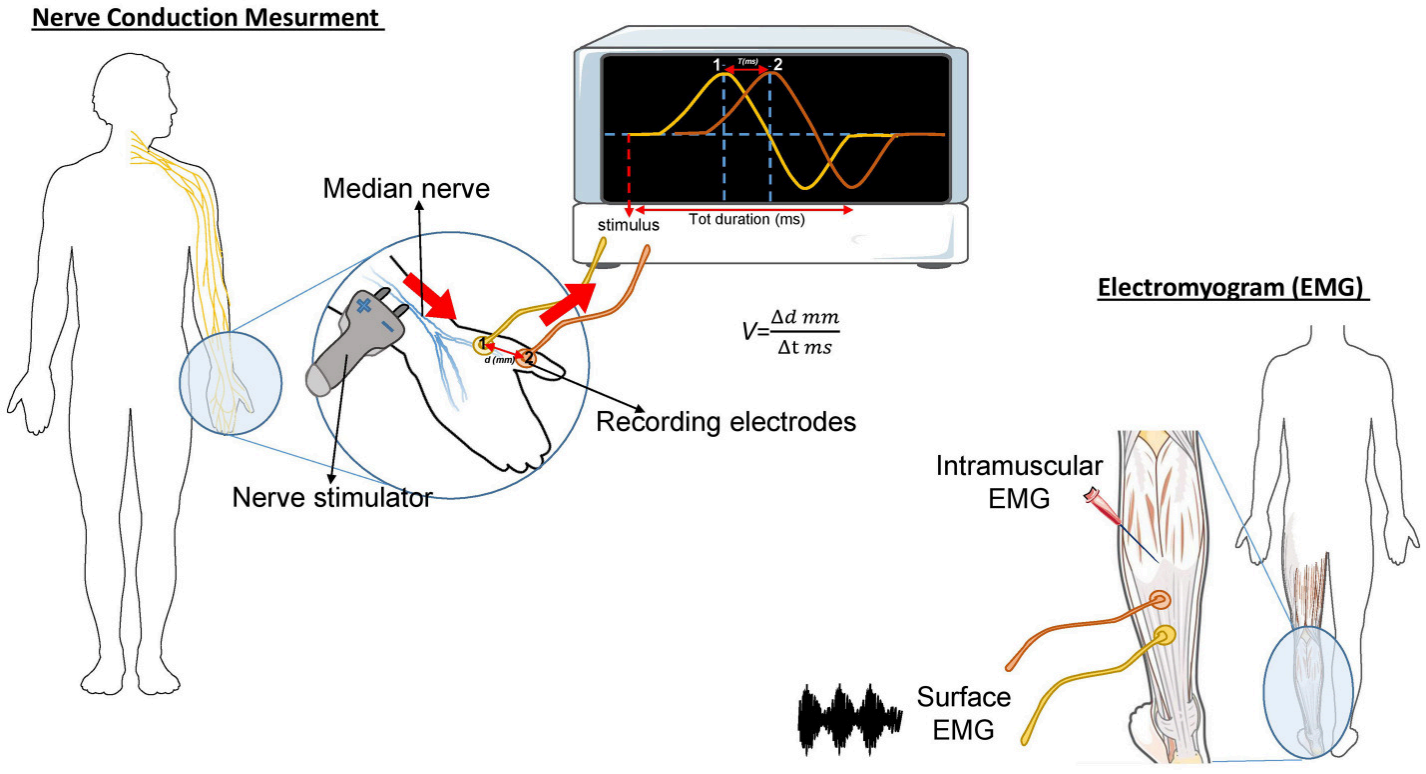
Schematic representation of the nerve conduction and muscle contraction studies. *Nerve Conduction Velocity (NCV - left)* measures the velocity and the quality of conduction of the electrical signal in a nerve. During the test, your nerve is stimulated, with an electrode attached to your skin. One or two more electrodes patches are placed on the skin over your nerve. The electrical impulse of the stimulated nerve pass from the stimulator to the other receiving electrode. The time (in milliseconds) spent by the impulse to move from a point to another, on the order of millimeters, represent the Velocity. In ALS, the impulse conduction is slower respect with control cases and is worsened by the increase of axonal degeneration. The *electromyogram (EMG-right)* measures the electrical activity of the muscles at rest and during contraction. There are two kinds of EMG: surface EMG and intramuscular EMG. In the first one the muscle activity is recorded by one or more electrodes patched on the skin and it asses the contractile response of superficial muscles. This approach presents several limitation since the result signal is influenced by the depth of the subcutaneous tissue at the site of the recording and by the discharges of adjacent muscles. With the intramuscular EMG, specific deep muscle activity is recorded by using one needle electrode inserted into the muscle. EMG and NCV tests are often done together to give more complete information.

The needle EMG is the most important study in determining diagnostic certainty of ALS (46). During this test, a needle electrode is inserted through the skin into various muscles, starting with the most severely involved limb (Figure 1). The examination then progresses through four anatomical region: bulbar, cervical, thoracic, and lumbar. At least three anatomical regions have to be positive to this test to define ALS. The fasciculation potential (FP) has been included in Awaji criteria as a hallmark of ALS muscular denervation. In general, a decreased number of motor unit recruitment, with long duration of the motor unit potential, and abnormal spontaneous activity, are measured at the EMG in ALS patients [reviewed in (47)].

#### Nerve Conduction Study (NCS)

This test measures how fast an electrical impulse moves through the nerve (Figure 1). During the test, one electrode placed on the skin stimulates the nerve of interest with a very mild electrical impulse. Variations in time spent to reach a second electrode can help in identifying a nerve damage. Whereas, EMG measures the electrical activity in the muscles, the nerve conduction study is specific for nerves and helps to localize the disorder among nerve, neuromuscular junction, and muscle. NCS is a powerful tool to discriminate ALS from axonal demyelination or conduction block impairments (48). NCS parameters are generally normal in ALS, albeit the presence of prolonged distal motor latency and slowed conduction velocity could be consistent with the diagnosis of ALS (49, 50). These changes suggest loss of large myelinated fibers, but also motor axons regeneration phenomena (50).

#### Magnetic Resonance Imaging (MRI)

This technique is able to produce detailed images of the brain and spinal cord, the latter with the advantage of simultaneously investigating the upper and lower motor neurons. During several years, its application was related to the exclusion of other disorders, as tumors or hernias that can display certain of the ALS-mimic symptoms (51). The evolution and improvement of this multimodal tool has recently become essential for the diagnosis of ALS. MRI scans can show cerebral degeneration and gray/white matter atrophy [reviewed in (52)], and also detect abnormalities in ALS muscle, likely due to denervation atrophy process (53).

#### Blood and Urine Tests

Testing hematological factors is helpful to exclude diseases that are capable of mimicking ALS symptoms. Recently, a population-based study, proposed serum albumin, creatinine levels, and lymphocyte count as markers for ALS, indicating muscle waste and inflammation respectively (54). Other markers potentially related to a better ALS outcome have been proposed: LDL/HDL levels, which are elevated in ALS plasma and represent a general unexplained hypermetabolism (55, 56); serum uric acid levels, which are decreased among ALS patients, further demonstrating the possible role of oxidative stress in the induction and propagation of the disease (57); serum ferritin levels which are elevated in ALS patients and could reflect perturbation in iron metabolism (58); concentrations of certain amino acids, which are decreased in ALS (59); levels of serum proinflammatory cytokines, such as IL-6, which are increased in ALS (60). Finally, high level of circulating AChE and metalloproteinases (MMP) have been reported in ALS plasma (61, 62) and although the exact source of these two classes of enzymes remains uncertain, it could in part reflect a disruption of extracellularly bound AChE at the NMJ and early change in the nerve-muscle integrity.

#### Spinal Tap (Lumbar Puncture)

Using this particular test, a small amount of cerebrospinal fluid (CSF) is taken from the lower back of the patient for laboratory tests. Thanks to its proximity to the central nervous system, the CSF is considered one preferred tissue to search for ALS biomarkers [reviewed in (63)]. Several markers for ALS have been identified in CSF such as Tau, TDP43, Nefl, and MMP levels [reviewed in (64)]. In particular, MMPs with their ability to digest collagen, proteoglycan, and laminin (65), may reflect ongoing destruction of the matrix which wraps synapses (66) and pathological changes at the brain-blood barrier (62).

#### Muscle Biopsy

With this technique, a small portion of muscle is removed by needle biopsy and sent to a laboratory for histopathological analysis. Rarely performed because of its painful and invasive nature, this tool is useful when ALS diagnosis is in doubt. Generally, ALS muscles present signs of active denervation/reinnervation and an increased number of atrophic fibers (67).

#### Genetic Testing

People with familial ALS (fALS) background can get an efficient diagnosis through genetic testing (68, 69). This technique may help ALS patients to understand the basis of their condition, and improve the genotype-specific treatments (70). Unluckily, there is a lack of consensus among clinicians above the definition of fALS, since newly genes related to ALS are continuously found (71). Nowadays, genetic testing is not wildly used because of its high cost and the belief that ALS genetics is not well-enough understood to provide a better treatment plan, as reported in 2017 in a study which involved 167 clinicians from 21 different countries around the world (71).

## Examples of NMJ Pathologies ALS-mimic

Here we report some examples of ALS-mimic pathologies. The *Spinal muscular atrophy (SMA)* is an inherited MND that prevalently affects children. Its incidence is 1 per 11,000 live births (72). All forms of SMA are caused by the loss of SMN1, a gene implicated in axonal mRNA transport and snRNP biogenesis (73). Studies involving mice and fly mutants demonstrated that the probable origin of this pathology resides in the early loss of sensory information from proprioceptive neurons (74), which in turn causes degeneration of α motor neurons. In consequence, progressive muscle weakness, and, in severe cases, respiratory failure appear (75). Despite being considered a child's illness, the SMA type 4, that has an adult onset, overlaps with ALS diagnosis (76). Furthermore, like in ALS, several studies reveal the early implication of NMJs in SMA, with synaptic pathology prior to the appearance of clinical symptoms (77–81). However, the first evidence of neuromuscular pathology occurred at different time points of the disease progression, with presynaptic pathology preceding morphological changes at the endplate in ALS, and simultaneous pre and post-synaptic pathologies in SMA, suggesting the possibility to study this particular zone in diagnosis (81). Histochemical skin biopsy comparison was suggested as a powerful diagnostic tool in differentiating ALS and SMA, since the small collagen fibrils and the increased amount of amorphous material, which are characteristic of ALS, are not in SMA (82).

The *Spinobulbar muscular atrophy [(SBMA) Kennedy's disease]* is a X-linked hereditary lower motor neuron disease, where the expanded trinucleotide repeat (CAG > 37) in the androgen receptor gene (AR) causes its nuclear inclusions and impairment of its function (83, 84). The disease affects 1 per 200,000 males in Europe and Asia each year (85). In this pathology, degeneration of anterior horn cells of the spinal cord, where androgen receptors are widely expressed, is observed (86, 87). Although SBMA patients exhibit facial weakness as first sign of the disease, they progressively develop myopathic features, such as muscle atrophy and necrotic myofibers (88). Like ALS, SBMA disease reveals mixed pathological findings, with both myopathy and neurogenic atrophy features, which is the cause of misdiagnosis at the early stage of the disease.

Among the autoimmune syndromes, *myasthenia gravis (MG)* overlaps ALS syndrome. The annual incidence ranges from 3 to 30 per 1,000,000 people (89). In fact, the binding of autoantibodies to components of the NMJ in MG causes a characterized muscle weakness and fatigability (90). Even if acetylcholine receptor antibodies are considered to be highly specific for the diagnosis of MG, ALS patients can also present these autoantibodies at the blood test (91, 92). In these cases, it is very difficult to define the false positive cases and an experimental treatment with AChE inhibitors is necessary to differentiate MG from ALS (93).

The skeletal muscle disorders are represented with the term *Myopathies*. Myopathies hold a list of pathologies (Table 1) where muscle weakness can begin in the hands and feet (distal muscles) as well as in the muscles near the center of the body (proximal muscles) sometimes mimicking ALS features, confusing the diagnostic and the treatment decision. Among them, *Inclusion Body Myositis (IBM)* (94) is the most common ALS-mimic disease. It is the most common adult myopathy in 50 year-old persons and older, and its incidence is 3.5 per 100,000 (95). It is characterized by inflammatory cells surrounding and invading non-necrotic muscle fibers, rimmed vacuoles, congophilic inclusions, and protein aggregates in muscle (96, 97). In this case, the unique way to exclude ALS is the muscle biopsy combined with quantitative electromyographic analysis, especially in those patients where disease progression is slow and atypical (98).

## Conclusion

Amyotrophic Lateral Sclerosis (ALS) and MNDs are not yet curable. However, accurate diagnosis is crucial to provide adequate counseling and information about the prognosis and disease course, and to avoid inappropriate therapy. Moreover, a good diagnosis could furnish a more equal stratification of cases and be important in the choice of additional medical support, as for example nutritional intervention strategies or physical therapy.

Currently, there is not a common consensus in the use of laboratory analysis for ALS diagnosis. Basically, clinicians decide for the application of certain techniques based on their experience, expertise and hospital practice. Progress in molecular genetics and identification of specific biomarkers is ongoing, which will translate to a refined diagnostic certitude. Therefore, there is the emerging need to establish a widely accepted protocol for laboratory tests to discriminate the majority of cases that present clinical features resembling ALS.

Increasing human and animal evidence proposed NMJ impairments as possible biomarkers for detection and discrimination of ALS and mimic diseases in an early, preclinical stage. However, further studies are needed to understand how these impairments could be monitored and specifically treated.

## Author Contributions

M-LC and A-RB contributed to conception and drafting. EK provided useful comments and reviewed the manuscript.

### Conflict of Interest Statement

The authors declare that the research was conducted in the absence of any commercial or financial relationships that could be construed as a potential conflict of interest.

## References

[1] Logroscino G Traynor BJ Hardiman O Chiò A Mitchell D Swingler RJ. Incidence of amyotrophic lateral sclerosis in Europe. J Neurol Neurosurg Psychiatry (2010) 81:385–90. 10.1136/jnnp.2009.183525 19710046 PMC2850819

[2] Chiò A Logroscino G Hardiman O Swingler R Mitchell D Beghi E. Prognostic factors in ALS: a critical review. Amyotroph Lateral Scler. (2009) 10:310–23. 10.3109/17482960802566824 19922118 PMC3515205

[3] Kiernan MC1 Vucic S Cheah BC Turner MR Eisen A Hardiman O. Amyotrophic lateral sclerosis. Lancet (2011) 377:942–55. 10.1016/S0140-6736(10)61156-7 21296405

[4] Morita M Al-Chalabi A Andersen PM Hosler B Sapp P Englund E. A locus on chromosome 9p confers susceptibility to ALS and frontotemporal dementia. Neurology (2006) 66:839–44. 10.1212/01.wnl.0000200048.53766.b4 16421333

[5] Vance C Al-Chalabi A Ruddy D Smith BN Hu X Sreedharan J. Familial amyotrophic lateral sclerosis with frontotemporal dementia is linked to a locus on chromosome 9p13.2-21.3. Brain (2006) 129:868–76. 10.1093/brain/awl030 16495328

[6] Robberecht W Philips T. The changing scene of amyotrophic lateral sclerosis. Nat Rev Neurosci. (2013) 14:248–64. 10.1038/nrn3430 23463272

[7] Strong MJ Hudson AJ Alvord WG. Familial amyotrophic lateral sclerosis, 1850-1989 : a statistical analysis of the world literature. Can J Neurol Sci. (1991) 18:45–58. 2036615 10.1017/s0317167100031280

[8] Brown RH Al-Chalabi A Amyotrophic lateral sclerosis. N Engl J Med. (2017) 377:162–72. 10.1056/NEJMra1603471 28700839

[9] Freischmidt A Wieland T Richter B Ruf W Schaeffer V Müller K. Haploinsufficiency of TBK1 causes familial ALS and fronto-temporal dementia. Nat Neurosci. (2015) 18:631–6. 10.1038/nn.4000 25803835

[10] Brenner D Müller K Wieland T Weydt P Böhm S Lulé D. NEK1 mutations in familial amyotrophic lateral sclerosis. Brain (2016) 139:e28. 10.1093/brain/aww033 26945885

[11] Mackenzie IR Nicholson AM Sarkar M Messing J Purice MD Pottier C. TIA1 mutations in amyotrophic lateral sclerosis and frontotemporal dementia promote phase separation and alter stress granule dynamics. Neuron (2017) 95:808–16.e9. 10.1016/j.neuron.2017.07.025 28817800 PMC5576574

[12] Rosen DR Siddique T Patterson D Figlewicz DA Sapp P Hentati A. Mutations in Cu/Zn superoxide dismutase gene are associated with familial amyotrophic lateral sclerosis. Nature (1993) 362:59–62. 10.1038/362059a0 8446170

[13] Renton AE Majounie E Waite A Simón-Sánchez J Rollinson S Gibbs JR. A hexanucleotide repeat expansion in C9ORF72 is the cause of chromosome 9p21-linked ALS-FTD. Neuron (2011) 72:257–68. 10.1016/j.neuron.2011.09.010 21944779 PMC3200438

[14] Sreedharan J Blair IP Tripathi VB Hu X Vance C Rogelj B. TDP-43 mutations in familial and sporadic amyotrophic lateral sclerosis. Science (2008) 319:1668–72. 10.1126/science.1154584 18309045 PMC7116650

[15] Kwiatkowski TJ Bosco DA Leclerc AL Tamrazian E Vanderburg CR Russ C Mutations in the FUS/TLS gene on chromosome 16 cause familial amyotrophic lateral sclerosis. Science (2009) 547:1205–9. 10.1126/science.1166066 19251627

[16] Nassif M Woehlbier U Manque PA. The enigmatic role of C9ORF72 in autophagy. Front Neurosci. (2017) 11:442. 10.3389/fnins.2017.00442 28824365 PMC5541066

[17] Bunton-Stasyshyn RKA Saccon RA Fratta P Fisher EMC. SOD1 Function and its implications for amyotrophic lateral sclerosis pathology: new and renascent themes. Neuroscientist (2015) 21:519–29. 10.1177/1073858414561795 25492944

[18] Guerrero EN Wang H Mitra J Hegde PM Stowell SE Liachko NF TDP-43/FUS in motor neuron disease: complexity and challenges. Prog Neurobiol. (2016) 145–6:78–97. 10.1016/j.pneurobio.2016.09.004 PMC5101148 27693252

[19] Dadon-Nachum M Melamed E Offen D. The ‘dying-back' phenomenon of motor neurons in ALS. J Mol Neurosci. (2011) 43:470–477. 10.1007/s12031-010-9467-1 21057983

[20] Campanari M-L García-Ayllón M-S Ciura S Sáez-Valero J Kabashi E. Neuromuscular junction impairment in amyotrophic lateral sclerosis: reassessing the role of acetylcholinesterase. Front Mol Neurosci. (2016) 9:160. 10.3389/fnmol.2016.00160 28082868 PMC5187284

[21] Cappello V Francolini M. Neuromuscular junction dismantling in amyotrophic lateral sclerosis. Int J Mol Sci. (2017) 18:E2092. 10.3390/ijms18102092 28972545 PMC5666774

[22] Dupuis L Loeffler J-P. Neuromuscular junction destruction during amyotrophic lateral sclerosis: insights from transgenic models. Curr Opin Pharmacol. (2009) 9:341–6. 10.1016/j.coph.2009.03.007 19386549

[23] Fischer LR Culver DG Tennant P Davis AA Wang M Castellano-Sanchez A. Amyotrophic lateral sclerosis is a distal axonopathy: evidence in mice and man. Exp Neurol. (2004) 185:232–40. 10.1016/j.expneurol.2003.10.004 14736504

[24] Frey D Schneider C Xu L Borg J Spooren W Caroni P. Early and selective loss of neuromuscular synapse subtypes with low sprouting competence in motoneuron diseases. J Neurosci. (2000) 20:2534–42. 10.1523/JNEUROSCI.20-07-02534.2000 10729333 PMC6772256

[25] Jokic N Gonzalez de Aguilar JL Pradat PF Dupuis L Echaniz-Laguna A Muller A. Nogo expression in muscle correlates with amyotrophic lateral sclerosis severity. Ann Neurol. (2005) 57:553–6. 10.1002/ana.20420 15786457

[26] Bruneteau G Bauché S Gonzalez de Aguilar JL Brochier G Mandjee N Tanguy ML. Endplate denervation correlates with Nogo-A muscle expression in amyotrophic lateral sclerosis patients. Ann Clin Transl Neurol. (2015) 2:362–72. 10.1002/acn3.179 25909082 PMC4402082

[27] Jokic N Gonzalez de Aguilar JL Dimou L Lin S Fergani A Ruegg MA. The neurite outgrowth inhibitor Nogo-A promotes denervation in an amyotrophic lateral sclerosis model. EMBO Rep. (2006) 7:1162–7. 10.1038/sj.embor.7400826 17039253 PMC1679784

[28] Clark JA Southam KA Blizzard CA King AE Dickson TC. Axonal degeneration, distal collateral branching and neuromuscular junction architecture alterations occur prior to symptom onset in the SOD1 G93A mouse model of amyotrophic lateral sclerosis. J Chem Neuroanat. (2016) 76(Pt A):35–47. 10.1016/j.jchemneu.2016.03.003 27038603

[29] Feiguin F Godena VK Romano G D'Ambrogio A Klima R Baralle FE. Depletion of TDP-43 affects Drosophila motoneurons terminal synapsis and locomotive behavior. FEBS Lett. (2009) 583:1586–92. 10.1016/j.febslet.2009.04.019 19379745

[30] Diaper DC Adachi Y Sutcliffe B Humphrey DM Elliott CJ Stepto A. Loss and gain of Drosophila TDP-43 impair synaptic efficacy and motor control leading to age-related neurodegeneration by loss-of-function phenotypes. Hum Mol Genet. (2013) 22:1539–57. 10.1093/hmg/ddt005 23307927 PMC3605831

[31] Sasayama H Shimamura M Tokuda T Azuma Y Yoshida T Mizuno T. Knockdown of the Drosophila fused in sarcoma (FUS) homologue causes deficient locomotive behavior and shortening of motoneuron terminal branches. PLoS ONE (2012) 7:e39483. 10.1371/journal.pone.0039483 22724023 PMC3378546

[32] Machamer JB Collins SE Lloyd TE. The ALS gene FUS regulates synaptic transmission at the Drosophila neuromuscular junction. Hum Mol Genet. (2014) 23:3810–22. 10.1093/hmg/ddu094 24569165 PMC4065154

[33] Kabashi E Lin L Tradewell ML Dion PA Bercier V Bourgouin P. Gain and loss of function of ALS-related mutations of TARDBP (TDP-43) cause motor deficits *in vivo*. Hum Mol Genet. (2009) 19:671–83. 10.1093/hmg/ddp534 19959528

[34] Kabashi E Bercier V Lissouba A Liao M Brustein E Rouleau GA Fus and tardbp but not sod1 interact in genetic models of amyotrophic lateral sclerosis. PLoS Genet. (2011) 7:e1002214 10.1371/journal.pgen.1002214 21829392 PMC3150442

[35] Armstrong GAB Drapeau P. Loss and gain of FUS function impair neuromuscular synaptic transmission in a genetic model of ALS. Hum Mol Genet. (2013) 22:4282–92. 10.1093/hmg/ddt278 23771027

[36] Ciura S Lattante S Le Ber I Latouche M Tostivint H Brice A. Loss of function of C9orf72 causes motor deficits in a zebrafish model of amyotrophic lateral sclerosis. Ann Neurol. (2013) 74:180–7 10.1002/ana.23946 23720273

[37] Van Damme P Robberecht W Van Den Bosch L Modelling amyotrophic lateral sclerosis: progress and possibilities. Dis Model Mech. (2017) 10:537–49. 10.1242/dmm.029058 28468939 PMC5451175

[38] Ghasemi M. Amyotrophic lateral sclerosis mimic syndromes. Iran J Neurol (2016) 15:85–91. 27326363 PMC4912674

[39] Traynor BJ Codd MB Corr B Forde C Frost E Hardiman O. Amyotrophic lateral sclerosis mimic syndromes. Arch Neurol. (2000) 57:109–13. 10.1001/archneur.57.1.109 10634456

[40] Brooks BR Miller RG Swash M Munsat TL. El Escorial revisited: revised criteria for the diagnosis of amyotrophic lateral sclerosis. Amyotroph Lateral Scler. (2000) 1:293–9. 10.1080/146608200300079536 11464847

[41] Miller RG Munsat TL Swash M Brooks BR. Consensus guidelines for the design and implementation of clinical trials in ALS. World Federation of Neurology committee on Research. J Neurol Sci. (1999) 169:2–12. 10.1016/S0022-510X(99)00209-9 10540001

[42] Traynor BJ Codd MB Corr B Forde C Frost E Hardiman OM. Clinical features of amyotrophic lateral sclerosis according to the El Escorial and airlie house diagnostic criteria. Arch. Neurol. (2000) 57:1171–6. 10.1001/archneur.57.8.1171 10927797

[43] De Carvalho M Swash M Awaji diagnostic algorithm increases sensitivity of El Escorial criteria for ALS diagnosis. Amyotroph Lateral Scler. (2009) 10:53–57. 10.1080/17482960802521126 18985466

[44] Haverkamp LJ Appel V Appel SH. Natural history of amyotrophic lateral sclerosis in a database population. Validation of a scoring system and a model for survival prediction. Brain (1995) 118(Pt 3):707–19. 7600088 10.1093/brain/118.3.707

[45] Davenport RJ Swingler RJ Chancellor AM Warlow CP. Avoiding false positive diagnoses of motor neuron disease: lessons from the Scottish Motor Neuron Disease Register. Neurol Neurosurg Psychiatry (1996) 60:147–51. 8708642 10.1136/jnnp.60.2.147 PMC1073793

[46] Daube JR. Electrophysiologic studies in the diagnosis and prognosis of motor neuron diseases. Neurol Clin. (1985) 3:473–93. 10.1016/S0733-8619(18)31017-X 3900681

[47] Joyce NC Carter GT. Electrodiagnosis in persons with amyotrophic lateral sclerosis. PM R (2013) 5:S89–95. 10.1016/j.pmrj.2013.03.020 23523708 PMC4590769

[48] Duleep A Shefner J. Electrodiagnosis of motor neuron disease. Phys Med Rehabil Clin N Am. (2013) 24:139–5. 10.1016/j.pmr.2012.08.022 23177036

[49] Argyriou AA Polychronopoulos P Talelli P Chroni E. F wave study in amyotrophic lateral sclerosis: assessment of balance between upper and lower motor neuron involvement. Clin Neurophysiol. (2006) 117:1260–5. 10.1016/j.clinph.2006.03.002 16678483

[50] de Carvalho M Swash M Nerve conduction studies in amyotrophic lateral sclerosis. Muscle Nerve (2000) 23:344–52. 10.1002/(SICI)1097-4598(200003)23:3<344::AID-MUS5>3.0.CO;2-N 10679710

[51] Filippi M Agosta F Abrahams S Fazekas F Grosskreutz J Kalra S. EFNS guidelines on the use of neuroimaging in the management of motor neuron diseases. Eur J Neurol. (2010) 17:526–e20. 10.1111/j.1468-1331.2010.02951.x 20136647 PMC3154636

[52] Turner MR Modo M. Advances in the application of MRI to amyotrophic lateral sclerosis. Expert Opin Med Diagn. (2010) 4:483–96. 10.1517/17530059.2010.536836 21516259 PMC3080036

[53] Staff NP Amrami KK Howe BM. Magnetic resonance imaging abnormalities of peripheral nerve and muscle are common in amyotrophic lateral sclerosis and share features with multifocal motor neuropathy. Muscle Nerve (2015) 52:137–9. 10.1002/mus.24630 25736373 PMC4474748

[54] Chiò A Calvo A Bovio G Canosa A Bertuzzo D Galmozzi F. Amyotrophic lateral sclerosis outcome measures and the role of albumin and creatinine. JAMA Neurol. (2014) 71:1134–42. 10.1001/jamaneurol.2014.1129 25048026

[55] Dupuis L Corcia P Fergani A Gonzalez De Aguilar JL Bonnefont-Rousselot D Bittar R. Dyslipidemia is a protective factor in amyotrophic lateral sclerosis. Neurology (2008) 70:1004–9. 10.1212/01.wnl.0000285080.70324.27 18199832

[56] Dorst J Kühnlein P Hendrich C Kassubek J Sperfeld AD Ludolph AC. Patients with elevated triglyceride and cholesterol serum levels have a prolonged survival in amyotrophic lateral sclerosis. J Neurol. (2011) 258:613–7. 10.1007/s00415-010-5805-z 21128082

[57] Keizman D Ish-Shalom M Berliner S Maimon N Vered Y Artamonov I. Low uric acid levels in serum of patients with ALS: Further evidence for oxidative stress? J Neurol Sci. (2009) 285:95–9. 10.1016/j.jns.2009.06.002 19552925

[58] Qureshi M Brown RH Rogers JT Cudkowicz ME. Serum ferritin and metal levels as risk factors for amyotrophic lateral sclerosis. Open Neurol J. (2008) 2:51–4. 10.2174/1874205X00802010051 19452011 PMC2627516

[59] Ilzecka J Stelmasiak Z Solski J Wawrzycki S Szpetnar M. Plasma amino acids concentration in amyotrophic lateral sclerosis patients. Amino Acids (2003) 25:69–73. 10.1007/s00726-002-0352-2 12836061

[60] Ono S Hu J Shimizu N Imai T Nakagawa H. Increased interleukin-6 of skin and serum in amyotrophic lateral sclerosis. J Neurol Sci. (2001) 187:27–34. 10.1016/S0022-510X(01)00514-7 11440741

[61] Festoff BW Fernandez HL. Plasma and red blood cell acetylcholinesterase in amyotrophic lateral sclerosis. Muscle Nerve (1981) 4:41–7. 10.1002/mus.880040108 7231444

[62] Niebroj-Dobosz I Janik P Sokołowska B Kwiecinski H. Matrix metalloproteinases and their tissue inhibitors in serum and cerebrospinal fluid of patients with amyotrophic lateral sclerosis. Eur J Neurol. (2010) 17:226–31. 10.1111/j.1468-1331.2009.02775.x 19796283

[63] Barschke P Oeckl P Steinacker P Ludolph A Otto M. Proteomic studies in the discovery of cerebrospinal fluid biomarkers for amyotrophic lateral sclerosis. Expert Rev Proteomics (2017) 14:769–77. 10.1080/14789450.2017.1365602 28799854

[64] Shaw PJ Williams R. Serum and cerebrospinal fluid biochemical markers of ALS. Amyotroph Lateral Scler Other Motor Neuron Disord. (2000) 1(Suppl. 2):S61–7. 10.1080/14660820050515773-1 11464944

[65] Vincenti MP Brinckerhoff CE. Signal transduction and cell-type specific regulation of matrix metalloproteinase gene expression: can MMPs be good for you? J Cell Physiol. (2007) 213:355–64. 10.1002/jcp.21208 17654499

[66] Lim GP Backstrom JR Cullen MJ Miller CA Atkinson RD Tökés ZA. Matrix metalloproteinases in the neocortex and spinal cord of amyotrophic lateral sclerosis patients. J Neurochem. (1996) 67:251–9. 10.1046/j.1471-4159.1996.67010251.x 8666998

[67] Jensen L Jørgensen LH Bech RD Frandsen U Schrøder HD. Skeletal muscle remodelling as a function of disease progression in amyotrophic lateral sclerosis. Biomed Res Int. (2016) 2016:5930621. 10.1155/2016/5930621 27195289 PMC4852332

[68] Lattante S Rouleau GA Kabashi E. TARDBP and FUS mutations associated with amyotrophic lateral sclerosis: summary and update. Hum Mutat. (2013) 34:812–26. 10.1002/humu.22319 23559573

[69] Ciura S Sellier C Campanari M-L Charlet-Berguerand N Kabashi E. The most prevalent genetic cause of ALS-FTD, C9orf72 synergizes the toxicity of ATXN2 intermediate polyglutamine repeats through the autophagy pathway. Autophagy (2016) 12:1406–8. 10.1080/15548627.2016.1189070 27245636 PMC4968221

[70] Roggenbuck J Quick A Kolb SJ. Genetic testing and genetic counseling for amyotrophic lateral sclerosis: an update for clinicians. Genet Med. (2017) 19:267–74. 10.1038/gim.2016.107 27537704

[71] Vajda A McLaughlin RL Heverin M Thorpe O Abrahams S Al-Chalabi A. Genetic testing in ALS: a survey of current practices. Neurology (2017) 88:991–9. 10.1212/WNL.0000000000003686 28159885 PMC5333513

[72] Kolb SJ Kissel JT. Spinal muscular atrophy. Neurol Clin. (2015) 33:831–46. 10.1016/j.ncl.2015.07.004 26515624 PMC4628728

[73] Burghes AHM Beattie CE. Spinal muscular atrophy: why do low levels of survival motor neuron protein make motor neurons sick? Nat Rev Neurosci. (2009) 10:597–609. 10.1038/nrn2670 19584893 PMC2853768

[74] Fletcher EV Mentis GZ Motor circuit dysfunction in spinal muscular atrophy. Spinal Muscular Atrophy. (2017) 153–65. 10.1016/B978-0-12-803685-3.00009-4

[75] Crawford TO Pardo CA. The Neurobiology of childhood spinal muscular atrophy. Neurobiol Dis. (1996) 3:97–110. 10.1006/nbdi.1996.0010 9173917

[76] Farrar MA Kiernan MC. The genetics of spinal muscular atrophy: progress and challenges. Neurotherapeutics (2015) 12:290–302. 10.1007/s13311-014-0314-x 25413156 PMC4404441

[77] Cifuentes-Diaz C Nicole S Velasco ME Borra-Cebrian C Panozzo C Frugier T. Neurofilament accumulation at the motor endplate and lack of axonal sprouting in a spinal muscular atrophy mouse model. Hum Mol Genet. (2002) 11:1439–47. 10.1093/hmg/11.12.1439 12023986

[78] Kariya S Park GH Maeno-Hikichi Y Leykekhman O Lutz C Arkovitz MS. Reduced SMN protein impairs maturation of the neuromuscular junctions in mouse models of spinal muscular atrophy. Hum Mol Genet. (2008) 17:2552–69. 10.1093/hmg/ddn156 18492800 PMC2722888

[79] Murray LM Comley LH Thomson D Parkinson N Talbot K Gillingwater TH. Selective vulnerability of motor neurons and dissociation of pre- and post-synaptic pathology at the neuromuscular junction in mouse models of spinal muscular atrophy. Hum Mol Genet. (2008) 17:949–62. 10.1093/hmg/ddm367 18065780

[80] Ling KKY Gibbs RM Feng Z Ko C-P. Severe neuromuscular denervation of clinically relevant muscles in a mouse model of spinal muscular atrophy. Hum Mol Genet. (2012) 21:185–95. 10.1093/hmg/ddr453 21968514 PMC3235013

[81] Comley LH Nijssen J Frost-Nylen J Hedlund E. Cross-disease comparison of amyotrophic lateral sclerosis and spinal muscular atrophy reveals conservation of selective vulnerability but differential neuromuscular junction pathology. J Comp Neurol. (2016) 524:1424–42. 10.1002/cne.23917 26502195 PMC5063101

[82] Ono S Mannen T Toyokura Y. Differential diagnosis between amyotrophic lateral sclerosis and spinal muscular atrophy by skin involvement. J Neurol Sci. (1989) 91:301–10. 10.1016/0022-510X(89)90059-2 2769298

[83] Li M Miwa S Kobayashi Y Merry DE Yamamoto M Tanaka F. Nuclear inclusions of the androgen receptor protein in spinal and bulbar muscular atrophy. Ann Neurol. (1998) 44:249–54. 10.1002/ana.410440216 9708548

[84] Takeyama K Ito S Yamamoto A Tanimoto H Furutani T Kanuka H. Androgen-dependent neurodegeneration by polyglutamine-expanded human androgen receptor in Drosophila. Neuron (2002) 35:855–64. 10.1016/S0896-6273(02)00875-9 12372281

[85] Fischbeck KH. Kennedy disease. J Inherit Metab Dis. (1997) 20:152–8. 10.1023/A:1005344403603 9211187

[86] Diaz-Abad M Porter NC. Rapidly worsening bulbar symptoms in a patient with spinobulbar muscular atrophy. Neurol Int. (2013) 5:21. 10.4081/ni.2013.e21 24416485 PMC3883066

[87] MacLean HE Warne GL Zajac JD. Spinal and bulbar muscular atrophy: androgen receptor dysfunction caused by a trinucleotide repeat expansion. J Neurol Sci. (1996) 135:149–57. 10.1016/0022-510X(95)00284-9 8867071

[88] Cortes CJ Ling SC Guo LT Hung G Tsunemi T Ly L. Muscle expression of mutant androgen receptor accounts for systemic and motor neuron disease phenotypes in spinal and bulbar muscular atrophy. Neuron (2014) 82:295–307. 10.1016/j.neuron.2014.03.001 24742458 PMC4096235

[89] Casetta I Groppo E De Gennaro R Cesnik E Piccolo L Volpato S. Myasthenia gravis: a changing pattern of incidence. J Neurol. (2010) 257:2015–9. 10.1007/s00415-010-5651-z 20623298

[90] Tai H Cui L Guan Y Liu M Li X Huang Y. Amyotrophic lateral sclerosis and myasthenia gravis overlap syndrome: a review of two cases and the associated literature. Front Neurol. (2017) 8:218. 10.3389/fneur.2017.00218 28588549 PMC5439131

[91] Abbott RJ Holden D Currie S. False positive anti-acetylcholine receptor antibodies in motorneurone disease. Lancet (1986) 1:906–7. 10.1016/S0140-6736(86)91005-6 2870369

[92] Ashizawa T. False positive anti-acetylcholine receptor antibodies in motorneurone disease. Lancet (1986) 1:1272. 10.1016/S0140-6736(86)91408-X 2872411

[93] Gold R Hohlfeld R Toyka KV. Progress in the treatment of myasthenia gravis. Ther Adv Neurol Disord. (2008) 1:36–51. 10.1177/1756285608093888 21180568 PMC3002545

[94] Naddaf E Barohn RJ Dimachkie MM. Inclusion body myositis: update on pathogenesis and treatment. Neurotherapeutics (2018) 15:995–1005. 10.1007/s13311-018-0658-8 30136253 PMC6277289

[95] Dimachkie MM Barohn RJ Inclusion body myositis. Neurol Clin. (2014) 32:629–46. 10.1016/j.ncl.2014.04.001 25037082 PMC4115580

[96] Chahin N Engel AG. Correlation of muscle biopsy, clinical course, and outcome in PM and sporadic IBM. Neurology (2008) 70:418–24. 10.1212/01.wnl.0000277527.69388.fe 17881720

[97] Chilingaryan A Rison RA Beydoun SR. Misdiagnosis of inclusion body myositis: two case reports and a retrospective chart review. J Med Case Rep. (2015) 9:169. 10.1186/s13256-015-0647-z 26268316 PMC4533788

[98] Dabby R Lange DJ Trojaborg W Hays AP Lovelace RE Brannagan TH. Inclusion body myositis mimicking motor neuron disease. Arch Neurol (2001) 58:1253–6. 10.1001/archneur.58.8.1253 11493165

